# Prediction of influential nodes in social networks based on local communities and users’ reaction information

**DOI:** 10.1038/s41598-024-66277-6

**Published:** 2024-07-09

**Authors:** Rohollah Rashidi, Farsad Zamani Boroujeni, MohammadReza Soltanaghaei, Hadi Farhadi

**Affiliations:** 1 Department of Computer Engineering, Isfahan (Khorasgan) Branch, Islamic Azad University, Isfahan, Iran; 2 Department of Computer Engineering, Science and Research Branch, Islamic Azad University, Tehran, Iran

**Keywords:** Identification of social network leaders, Social network analysis, Identification of communities, User influence, Computational science, Computer science, Information technology

## Abstract

Identifying influential nodes is one of the basic issues in managing large social networks. Identifying influence nodes in social networks and other networks, including transportation, can be effective in applications such as identifying the sources of spreading rumors, making advertisements more effective, predicting traffic, predicting diseases, etc. Therefore, it will be important to identify these people and nodes in social networks from different aspects. In this article, a new method is presented to identify influential nodes in the social network. The proposed method utilizes the combination of users’ social characteristics and their reaction information to identify influential users. Since the identification of these users in the large social network is a complex process and requires high processing power and time, clustering and identifying communities have been used in the proposed method to reduce the complexity of the problem. In the proposed method, the structure of the social network is divided into its constituent communities and thus the problem of identifying influential nodes (in the entire network) turns into several problems of identifying an influential node (in each community). The suggested method for predicting the nodes first predicts the links that may be created in the future and then identifies the influential nodes based on an iterative strategy. The proposed algorithm uses the criteria of centrality and influence domain to identify this category of users and performs the identification process both at the community and network levels. The efficiency of the method has been evaluated using real databases and the results have been compared with previous works. The results demonstrate that the proposed method provides a more suitable performance in detecting the influential nodes and is superior in terms of accuracy, recall and processing time.

## Introduction

### Motivation

Social networks are an integral part of the world of communication. The problem of social network analysis is a very broad problem and the results obtained from it can be used in various research areas such as capital participation networks, scientific cooperation networks and fan networks, transportation networks, electricity distribution networks, etc.^1^. The range of use and universality of social networks has made it very important. It seems evident that in the use and management of a network with this importance and scope of application, various problems and challenges may emerge. One of these issues (if solved, the usefulness of social networks can be significantly improved) is the identification of influential users^2^. Based on the studies conducted in the field of identifying influential users in social networks, there seem two basic challenges that have not been addressed simultaneously by previous studies. First, the identification of influential users should be carried out according to the scope of their influence on other users^3^. For instance, the influence of a popular personality in the field of information technology may be insignificant to users interested in literature and art. As a result, considering this factor can be effective in improving the accuracy of diagnosis. Second, due to a large amount of information in social networks, analyzing the network and identifying influential users in a short time will be one of the most fundamental challenges^4^.

### Research background

Research records related to the proposed method can be followed in the two directions of identifying communities and identifying influential nodes in social networks. The proposed methods in^5,6^ use optimization problems to find influential users in social networks. These algorithms evaluate different solutions using evolutionary search and provide the best set of influential users as final results. The proposed method in^5^ performs this operation using the gray wolf optimization (GWO) algorithm, while the method presented in^6^ uses the multi-objective optimization algorithm to achieve this goal. In^7^, minimum spanning tree and modularity criteria are used to detect communities. In this research, first, the network graph is weighted using a node dissimilarity criterion. Then a minimum spanning tree is generated from the weighted graph. By removing connections that have a high amount of dissimilarity, complex network groups are obtained. In the next step, the groups are merged using the modularity criterion and by an action iterative algorithm. Zhao et al. in^8^ investigated the importance of the role of nodes in several different networks by using the indices of degree centrality, betweenness centrality, closeness centrality and semi-local centrality. Each of the centrality parameters in social networks plays a role. Each of the networks has special characteristics due to the nature of the relationship governing the network. For example, we can refer to the directed and non-directed nature of the graph, as well as the two-part graph and the absence of loops in social networks. Due to these differences in social networks, the presented indices and conclusions are not always true for all types of networks. It was found that for each environment, according to the type of problem, the way of spreading the influences and also the probability of affectability, the best criterion can be different. Vathi et al.^9^, by taking into account the characteristics of following, common followers and common friends from the structural aspect, hashtags from the content aspect and responses, retweets and mentions from the interactive aspect, present the similarity criteria and then identify communities using the affinity propagation algorithm. Analyzing these data can lead to the discovery of unknown information and relationships in these networks. Discovering a community consisting of “similar” nodes is an important challenge in the field of social network data analysis, and is widely important in the field of graph structure in these networks.

To identify communities, Mousavi et al.^10^, in addition to the structural similarity between users, paid attention to the degree of similarity between actions performed by users in social networks. In this method, a table of all actions performed by users is created, which shows what actions were performed by which users in the social network. Wang et al. in^11^ combined network graph structure and user profile features to identify overlapping social circles. In the proposed method, the structural similarity between users is first checked using the Salton index, and then the degree of similarity between users is analyzed in terms of the number of common features in the profile. Hu and Yang^12^ proposed a method in which the act of identifying user communities in the social network was performed by clustering the edges between users in the social network graph. In this way, for each pair of edges that have a common node at one end, the structural similarity is calculated using the Jaccard index, and if there is no common node between the two edges, the structural similarity is considered zero. Previous research majorly focused on identifying influential nodes in social networks and paid less attention to the subject of prediction. This identification is done based on criteria such as the number of followers, centrality, and the like. Although using these criteria, users who have more followers can be identified, this category of people may not have many roles in influencing other users; because based on the described content, the influence domain of each user will be limited^13^. In this situation, it seems that it is more appropriate to consider the reaction criteria of users as a basis for recognizing the effectiveness of people in the social network. This feature is considered the basis of current research. In Table 1, different methods are compared based on the parameters of prediction, trust and interactions.

**Table 1:** Taxonomy of recent research works.

References	Forecasting leader nodes	Trust	Degree	Interaction	Grouping	Dataset
^5^	No	No	Yes	No	No	Hamsterster, Pretty Good, Astro
^6^	No	No	Yes	No	No	WikiVote, NetHEPT, Epinions
^7^	No	No	Yes	No	Yes	Zachary’s karate club, College football network, Books
^8^	No	No	Yes	No	Yes	Football, SFI, Email, Facebook, PGP
^9^	No	No	Yes	Yes	yes	Twitter
^10^	No	No	Yes	No	Yes	Zachary’s karate club, Lusseau’s dolphins’ network, American college football teams
^11^	No	No				Email, Reality, Web, Brightkite
^12^	No	No	Yes	No	No	Email, Reality, Web, Brightkite
^13^	No	No	Yes	No	Yes	Small slashdot
Proposed model	Yes	Yes	Yes	Yes	Yes	Twitter-Dynamic-Net

### Research gaps and contributions

As per the analysis of various references, it seems that the authors mostly focus on agent-based systems or dynamic models to use for marketing and socio-economic modeling with consideration of trust. The meaning of trust is the detection of spam and the validity of offers^14^. Based on the literature, most of the research deals with identifying methods, discovering influential nodes or reducing the time of information dissemination by using different characteristics of nodes, and less research has focused on simultaneously discussing different indicators of the characteristics of nodes such as trusted, structure attribute, and interaction. There has been significant growth and development in the field of identifying the leader nodes with all their positive and negative aspects. However, determining which method is better according to various topics and situations is worth discussing. It is worth mentioning that many activities have been done in the field of identifying or discovering influential nodes, but little activity has been done in the field of predicting leader nodes based on the current future. What has been done in the field of prediction is mostly link prediction. In social and critical studies, we are looking for influential users who can solve problems with various issues, such as accelerating the change of a group’s behavior, increasing the probability of accepting new norms, or helping to spread a certain treatment. Considering the scalability and variety of data, statistical methods are inefficient for solving such issues. For such data, machine learning methods and topological measurements are suggested. Nonetheless, social networks contain different dimensions of data, including textual data, interactions between users, the importance of each node, the number of followers, etc. However, the problem with topological methods is that they cannot examine more than one network structure to determine the centrality of a node. On the other hand, the lack of rich data is the problem of the machine learning method. Therefore, to overcome the mentioned problems, it can be useful to use hybrid methods that can incorporate the concepts of network structure and statistical methods and machine learning. Many questions are worth to be addressed, including these questions: How to calculate the ability of a node to disseminate information? Which entities are important or influence other nodes and which nodes are followers? How were the groups formed and how will they be formed in the future? Which connections are important or which connections may be formed in the future? Which nodes accelerate the dissemination of information or prevent the dissemination of information? How reliable are these networks? How can these networks be used? How do distinguish reliable sources from unreliable sources?

The main idea of this article is to predict the selection of nodes as leader nodes at a certain point in time in the future using the available data collected at a certain time interval in the past. Because social networks are constantly changing, predictions are also changing over time. In other words, by having the network graph at the moment *t*, new nodes of the leader in the time interval from *t* to *t*′ are predicted in such a way that by using the parameters, we can predict the probability of selecting a leader node in the future or, in the graph, we calculate the probability of which node will be more interested in the future. Of course, our goal in this prediction is not the possibility of nodes becoming leaders in continuous intervals of time, but only the information of the previous year is used for possible prediction currently or in a limited period. The points that are taken into consideration in node prediction are the use of node interactions and content analysis of message text to identify malicious nodes and separate them from leader nodes as well as incorporating the concept of trust and information acquisition sources to identify the leader nodes. The main goal of the article is to predict the leader nodes. To achieve this goal, the parameters that can be effective in identifying the leader node are used. This work attempts to derive a model by sampling the processes that a node goes through to become an influential node, and based on that model, predict the node. To predict the leader node, link prediction is used as a strategy to adopt the links that will be added to the network in the future to predict the node.

The remainder of this article is organized as follows. In section “Methodology”, a proposed method for identifying influential users in social networks is presented. In section “Implementation and results”, the results of the implementation and evaluation of the proposed method are presented, and in section “Discussion and conclusion”, the conclusion is discussed.

## Methodology

The purpose of predicting leader nodes is to predict these nodes at a certain point in time in the future by using the available data collected at a certain time interval in the past. As a result of the ever-changing characteristics of social networks, projections are continuously subject to modification. To provide further clarification, the network graph is utilized to forecast the leader’s new nodes from time t to time t′. Through the application of the parameters, it is possible to approximate the probability of future leader node selection. Alternatively, the probability of which node will exhibit greater interest in the future can be computed using the graph as a guide. The aim of this forecast is not to make continuous predictions regarding the ascent of nodes to leadership positions over time; instead, it is to employ data from the previous year to generate potential predictions for the current or a specified time period. Node prediction involves the examination of various factors, including the analysis of message text and node interactions, in order to identify malicious nodes and differentiate them from leader nodes. Furthermore, the system utilizes sources of information acquisition and trust in order to ascertain the leader nodes.

In this section, we will describe the proposed algorithm to identify influential users in social networks. The proposed solution to identify this category of users includes four main steps:

1. Determining the value of network communication based on users’ reaction information to create a reliable network structure
2. Identification of communities in the social network structure
3. Link prediction based on the established reliable network
4. Calculation of centrality criteria for members of each community
5. Identifying influential nodes based on centrality and influence domain

**Figure 1:**
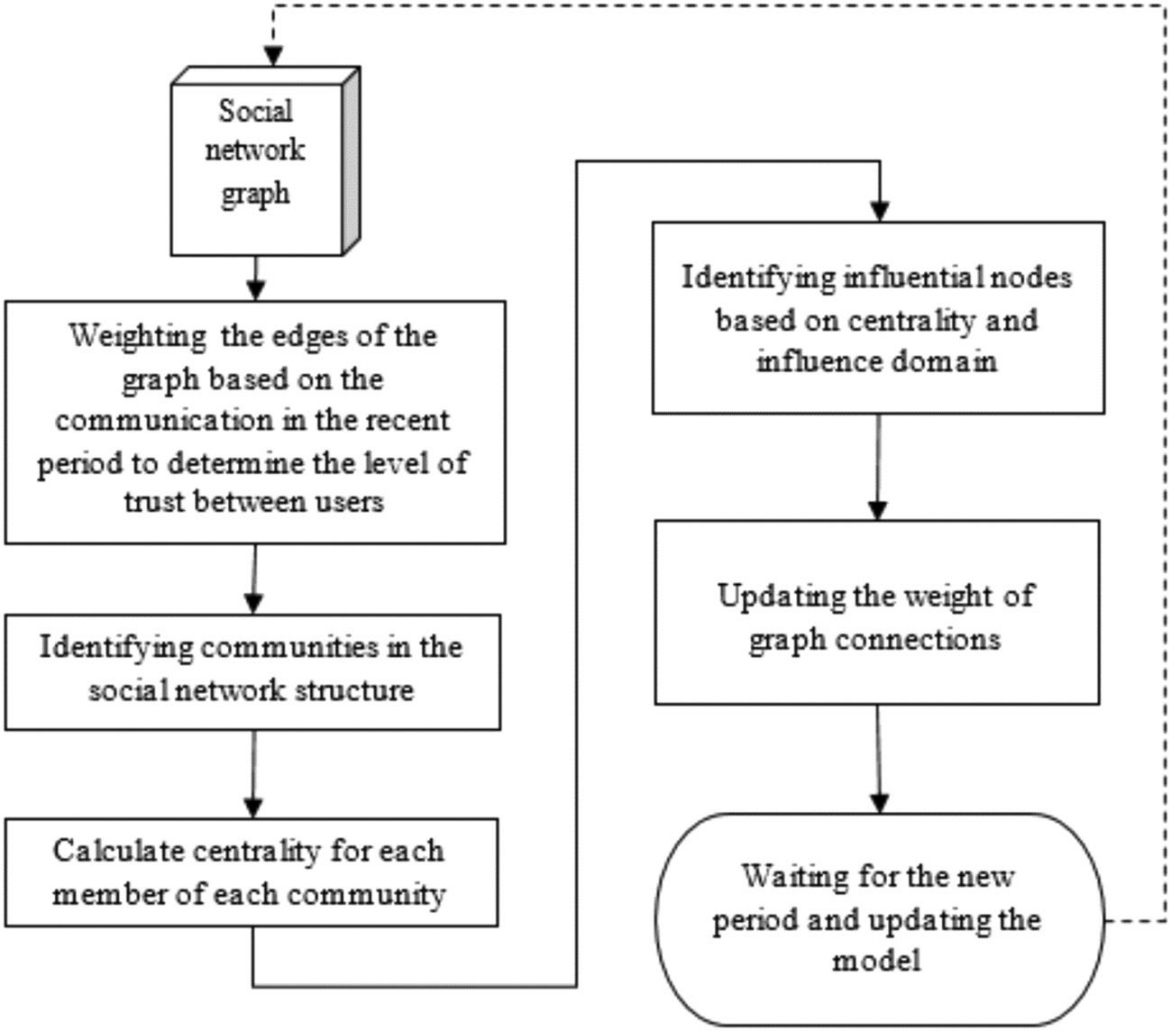
Diagram of steps of the proposed method.

The diagram of the steps of the proposed method is shown in Fig. 1

Based on the diagram shown in Fig. 1, the act of identifying the influential nodes is done based on specific periods. First, the social network is considered a directed graph and the users of this network are described as network nodes. The first step in the proposed method is to determine the importance of communication between users by calculating the weight of edges between network nodes. For this purpose, a criterion based on the rate of information dissemination and reactive behavior of users will be used. In the second step of the proposed method, the structure of the weighted network is decomposed into its constituent communities. The purpose of doing this is to reduce the complexity of the problem of identifying the influential nodes through their decomposition. After identifying the communities, centrality criteria were calculated for the members of each community to determine the importance of each node in its community. In the last step of the proposed method, an iterative algorithm is used to identify influential nodes based on the centrality and influence domain of the node. The result of this algorithm will be the collection of influential users in the global domain of the network and the local domain of communities. Since the events in social networks are based on time, the time of the events can be effective in determining the influential nodes. For this reason, in the proposed method, the influential nodes are identified sequentially and during specific time intervals. In the following, we will explain the details of each of these steps in the proposed method.

### Determining the value of network communication based on users’ reactive information to create a reliable network structure

One of the challenges of the proposed idea is to check the trust of nodes because one of the most basic aspects of social communities is the trust between the members of that network. Sherchan et al.^15^ examine various aspects of trust, including computational, relational, emotional, cognitive, etc., in social networks. Among them, the relational aspects of trust are most applicable in the context of social networks because trust is built based on several interactions between opinion leaders and followers. Reliability and dependency in previous interactions are two important factors in determining the reliability between two parts. In online systems, there are two types of trust, one is direct trust and the other is suggested trust, the former is based on direct experiences between two parties, and the second one is based on the suggestion of other members of the community. Among the research carried out in the field of social networks is the identification of users based on their characteristics; how these characteristics are extracted and how accurate and reliable they are very important and in achieving the goals of distinguishing trusted users from untrusted users. One of the weaknesses of centrality methods is that they neglect the reliability of nodes. In this research, we calculate the level of trust between two users based on the number of interactions between them.

We consider a social network described as a graph like *G*. This complex network is a directed and weighted graph. In this graph, users are considered as graph nodes and the relationship between users is displayed as a directed and weighted edge. The direction of each edge is from the followed user to the follower user and the weight of each edge will indicate the number of messages viewed by the follower user. As it is clear from the nature of social networks, all the connections between network users are not the same in terms of importance. One communication can specify the reaction to thousands of messages, while another communication only shows a reaction to a message. Therefore, in the proposed method, we describe the importance of each relationship in the graph using a weight criterion. For this purpose, a criterion called the probability of user reaction has been adopted. For example, the initial weight of a directed edge between two nodes A and B is equal to the number of reactions made by user B to the content provided by user A. In real social networks, the value of this connection in the social network graph is equal to the probability that user B reacts to a message sent by user A. Therefore, the normalized weight of the connection between two nodes in the social network can be calculated based on the reaction probability criterion as follows:

1 $$W_{ij} = \frac{1}{3}\left( { \frac{{f_{ij} }}{{\Sigma f_{i}^{G} }} + \frac{{L_{ij} }}{{\sum L_{i}^{G} }} + \frac{{C_{ij} }}{{\sum C_{i}^{G} }}} \right)$$

where, $W_{ij}$ specifies the weight of the connection between users $i$ and $j$ in graph* G*. Also, $f_{ij}$ represents the number of messages from user $i$ that have been republished by user $j$, and $\Sigma f_{i}^{G}$ specifies the total number of messages from user $i$ that have been republished by other users. Also, $L_{ij}$ and $C_{ij}$ respectively indicate the number of messages from user $i$ that have been liked and commented on by user $j$. Finally, $\sum L_{i}^{G}$ and $\sum C_{i}^{G}$ describe the total number of likes and comments for messages belonging to user $i$. In fact, the above equation describes the probability of each user’s reaction to the content produced by a specific user as a numerical value in the range (0, 1]. By applying Eq. (1) to all social network communications, a weight graph will be obtained that, in the future, will be used to identify communities and the level of trust.

### Identification of communities in the social network structure

The second step in the proposed method is to decompose the weighted graph obtained from the previous step into its constituent communities. This step is done to reduce the complexity of the problem of identifying influential nodes by decomposing the problem into smaller problems. We consider graph *G* with connections as weighted in Eq. (1). The weight of each communication is determined based on the probability of other users reacting to each user’s content. As a result, the lower value of the weight for each connection in the graph indicates the probability of a low reaction to the content produced by the user. In the proposed method, first, the weight values of network connections are reversed as follows^7^:

2 $$W_{ij}{\prime} = \frac{1}{{w_{ij} }}$$

After converting the weight values by the above equation, this criterion can be used to determine local communities so that by choosing connections with less weight, users with stronger connections will also be selected. To achieve this goal, the tree structure used in^7^ can be used to describe a graph. The tree structure means that each network user is examined only once. As a result, the computational complexity of the proposed method will be reduced. To identify the communities formed through the weighted graph, first, the minimum spanning tree corresponding to the weighted graph is created using the Prim algorithm. This action removes some of the communications with high weight (or in other words, the possibility of less reaction to the user’s content) from the network. The steps of forming the minimum spanning tree by Prim’s algorithm are as follows^7^:


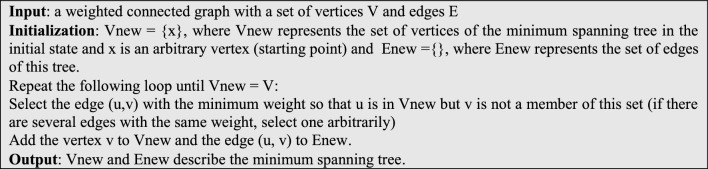


**Figure 2:**
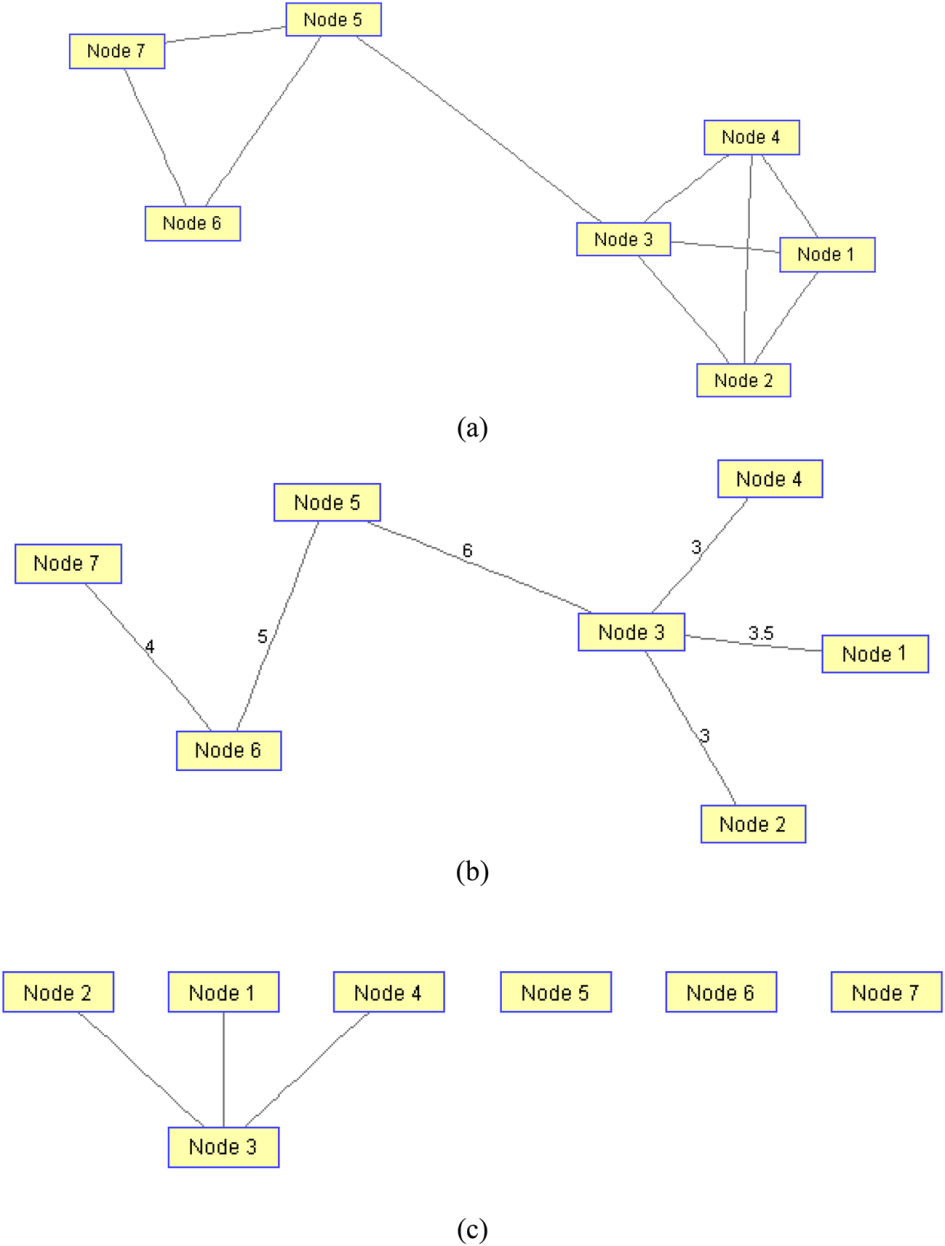
Steps to identify local communities (**a**) initial hypothetical graph, (**b**) graph weighting result and minimum spanning tree formation for the hypothetical graph, (**c**) initial local communities obtained by removing connections with higher weights for the hypothetical graph.

After forming the minimum spanning tree, the next step is to remove the edges with the highest weight in the tree. If the desired network has *N* nodes, $\left\lfloor {\frac{N - 1}{2} } \right\rfloor$ connections with higher weight in the obtained minimum spanning tree can be removed to form $\left\lfloor {\frac{N + 1}{2} } \right\rfloor$ local community. These local communities have the largest set of common neighborhoods within themselves because the connections of their internal edges have the lowest weights. The implementation steps of this part of the proposed method are shown on a hypothetical graph in Fig. 2.

The graph shown in Fig. 2a is a small network with 7 users. The aim is to identify the communities that are more likely to respond through the proposed method. For this, the local network communities must be identified through the steps mentioned. As mentioned, the first step is weighting the network graph. The result of graph weighting based on Eq. (2) and minimum spanning tree formation for the assumed graph is shown in Fig. 2b. To determine the local communities through the obtained minimum spanning tree, we remove the edges with more weight. Having 7 nodes in the assumed graph, $\left\lfloor {\frac{7 - 1}{2}} \right\rfloor = 3$ connections with higher weight will be removed and initial local communities will be formed. The result of this work is shown in Fig. 2c. By doing this, four local communities have been obtained for the assumed graph: lC(1) = {v1,v2,v3,v4}, lC(2) = {v5}, lC(3) = {v6} and lC(4) = {v7}.

The next step of the proposed method is to merge the obtained communities so that the final communities are produced. In the following, we will discuss the process of identifying communities using the proposed algorithm^7^. If lC(G) is the set of all local communities of G, the proposed algorithm uses an iterative approach to identify communities in the network. In this step, the local communities of the network are checked in pairs. In this step, a local fitting function is incorporated to do this, and communities that can achieve the greatest increase in modularity are merged. Modularity is a scale with a value in the range [− 1, 1] that measures the ratio of the density of communications within the community to communications outside the community. For a weighted graph, the modularity criterion is calculated as follows^13^:

3 $$Q = \frac{1}{2m}\mathop \sum \limits_{ij} \left[ {A_{ij} - \frac{{k_{i} k_{j} }}{2m}} \right]\delta (c_{i} \cdot c_{j} )$$

In the above relationship: A_ij_ indicates the existence of a connection between two vertices i and j. k_i_ and k_j_ represent the sum of weights of edges connected to nodes i and j, respectively. Also, *m* is the total weight of all edges of the graph, ci and cj are communities of network graph nodes, with nodes i and j as members of them, respectively. Finally, δ is the simple Kronecker delta function. In this function, if i = j then $\delta (c_{i} ,\;c_{j} ) = 1$ and otherwise $\delta (c_{i} ,\;c_{j} ) = 0$. In this step, a community is defined as a subgraph that is determined by maximizing the fitness property of the nodes. The best fitness for each graph segmentation is calculated using the following equation^14^:

4 $$f_{C} = \frac{{D_{in}^{c} }}{{\left( {D_{in}^{c} + D_{out}^{c} } \right)^{a} }}$$

In the above equation, $D_{in}^{c}$ and $D_{out }^{c}$ are the degrees of internal and external vertices of C, respectively, and parameter *a* is a positive variable that determines the size of the community. The degree of internal vertices is equal to twice the number of internal connections of part C and the degree of external vertices is equal to the number of edges that part C has with other parts of the network. Based on the above equation, the effect of the presence of node N on the fitness of section C can be calculated as follows:

5 $$f_{C} (N) = f_{C \cup N} - f_{C - N}$$

The term $C \cup N$ in the above equation indicates the fitness of the subgraph in the case N is its member, and $f_{C - N}$ indicates the fitness of the subgraph in the case N is not its member. In the proposed method, each local community is considered as a node like N in the above equations. The membership of node N in the subgraph C is done through the following iterative steps^7,13,14^:

1. Using an iteration loop, the neighboring nodes of subgraph C are examined as follows:
2. A neighboring local community of C such as N that has the highest fitness is added to C to obtain a subgraph such as C′.
3. The fitness of the new community C′ is recalculated.
4. If merging the local community N with C′ decreases the fitness value, it is removed from the subgraph C′.
5. Two local communities whose combination will achieve the greatest fitness are merged.
6. This process is repeated until no more communities can be integrated.

Considering this clustering structure for the social network, in the next step, the proposed method of different centrality criteria for each member in the structure of the communities is obtained from the social network, which will be described in the following subsections.

If the general society is not divided into sub-communities, we will not have an accurate forecast. So, the proposed process takes time, but the solution obtained is a more accurate and reliable. A lot should be spent on separating topics because the characteristics and features of different societies are different to predict influential nodes with different topics. On the other hand, the response time of the system is not very important in predicting the leader nodes.

### Link prediction based on the trusted network

After creating a network structure consisting of communities and reliable connections, FriendLink algorithm is used to predict the future communication between social network users. It should be noted that in this step, the reliable network obtained from the first step of the proposed method is adopted as the input of the link prediction algorithm. If social network users and connections between each pair of them are considered as nodes and edges, respectively, then it is possible to generate paths with more than one step between multiple users. To calculate user similarity based on link prediction and based on reliable paths with different lengths between users, the similarity matrix is updated. If v_x_ and v_y_ are two vertices of the graph, then the matrix of paths of lengths two and three can be produced under the condition that there are no duplicate vertices. The higher the number of these paths, the higher the probability of friendship. Therefore, assuming a matrix containing paths of lengths one and two for all pairs of network vertices, the similarity between two users v_x_ and v_y_ can be calculated as follows^3^:

6 $$sim(v_{x} \cdot v_{y} ) = \mathop \sum \limits_{i = 2}^{l} \frac{1}{i - 1} \cdot \frac{{\left| {path_{{v_{x} .v_{y} }}^{i} } \right|}}{{\mathop \prod \nolimits_{j = 2}^{i} (n - j)}}$$

In the above equation, *n* is the number of nodes in the graph and *l* is the maximum length considered for a path between two nodes v_x_ and v_y_. There can be no loop in these paths. $\frac{1}{i - 1}$ is a damping coefficient that weights paths of different lengths. For example, paths with a length of two are considered with a coefficient of $\frac{1}{2 - 1} = 1$; While paths with a length of 3 with a coefficient of $\frac{1}{3 - 1} = 0.5$ play a role in the relationship of similarity calculation. In the above relationship, $\left| {path_{{v_{x} .v_{y} }}^{i} } \right|$ is the number of all non-loop paths with length i between two nodes v_x_ and v_y_.

By applying the link prediction algorithm on pseudo-trusted users, a similarity matrix will be obtained that predicts the probability of communication between users based on their trusted connections. After forming the similarity matrix, the most probable calculated connection is added to the reliable network so that the leader nodes are predicted based on the obtained connections.

### Calculation of centrality criteria for members of each community

After identifying the clustering structure (communities) and predicting new links in social network communities, the centrality criteria of each node are calculated. This practice is used to identify influential nodes in communities. To calculate the centrality of each node, the following equation is used in^16^:

7 $$\theta_{x} = \frac{1}{4}\left( {CC(x) + LC(x) + DC(x) + PR(x)} \right)$$

where, the criteria CC, LC, DC and PR indicate closeness centrality, semi-local centrality, degree centrality and PageRank, respectively. In this section, the method of calculating each of these criteria is explained.

(A) *Closeness centrality*: This measure of centrality is based on radius and length. The most common criterion of centrality in this group is Freeman’s closeness centrality. Closeness centrality is the reciprocal of the average distance of a node to other nodes of the graph. The node with the highest value of closeness has more access to other nodes and can send information to all nodes or receive information from them in a short period. The closeness of node x is equal to the reciprocal of the average shortest distance of node x to other nodes of the graph. How long does it take for information from one node to reach other nodes (nodes that have access to it). It is suitable for finding the fastest publishing place. This criterion is calculated using the following equation^17^:

8 $$CC(i) = \frac{1}{{\mathop \sum \nolimits_{j \ne i} d(j \cdot i)}}$$

$d(j \cdot i)$ is the smallest distance between nodes i and j in the graph. This criterion can be calculated in the network if the entire network is connected. If there are nodes that do not have access to each other, their distance to other nodes will be infinite and this criterion will be zero for them and will be unusable. In this case, the efficiency criterion is used, which has a formula similar to closeness, but removes the limitation of the connectedness of the graph.

(B) *Semi-local centrality*: The second criterion for calculating the influence of the node is the semi-local centrality criterion of the node. To calculate the semi-local centrality, the nearest neighbors to the node and the neighbors of its neighbors are calculated and used in the centrality calculation. The calculation of semi-local centrality for a node like i is realized using the following equation^18^:

9 $$Q(u) = \mathop \sum \limits_{w \in \Gamma (u)} N(w)$$

10 $$LC(i) = \mathop \sum \limits_{u \in \Gamma (i)} Q(u)$$

where, Γ(u) is the set of neighbors of node u and N(w) is the number of nearest neighbors and next closest neighbors of node w. This method is less computationally complex than the global methods and in two steps the neighbors of w are obtained to calculate N(w).

(C) *Degree centrality*: The simplest and most common criterion of centrality is the i-degree centrality of the node or DC(i) in the adjacency matrix of the network. The degree of a node is the number of nodes that are directly adjacent to that node. The higher the degree of a node, the greater the importance of that node. This criterion is described as follows:

11 $$DC(i) = \deg (i) = \left| {\Gamma (i)} \right|$$

One approach to interpreting degree centrality is to count the number of paths of length one from a node.

(D) *PageRank centrality criterion*: This criterion was first developed by Breen and Page. It has been assumed that a random passenger follows the structure of a network by the transfer matrix P and sometimes randomly goes to another node in the network with a probability of $\frac{1}{n}$. Then the PageRank vector, which is an *n*-dimensional vector, will be calculated as follows using frequent updating^19^:

12 $$v^{t + 1} = \left( {\left( {1 - \beta } \right)P + e\frac{\beta }{n}} \right)v^{t}$$

This equation will be repeated until the following condition is met^19^:

13 $$E = \left| {v^{t + 1} - v^{t} } \right| = \mathop \sum \limits_{i = 1}^{n} (v_{i}^{t + 1} - v_{i}^{t} )^{2} < \epsilon$$

The initial value of vector *v*, which is the initial PageRank, is considered $\frac{1}{{\text{n}}}$ for all nodes. *e* is the unit matrix and *β* is the restart probability. Also, we have set β = 0.15 and $\varepsilon = 10^{ - 3}$. Matrix *P* is also defined as follows^19^:

14 $$P_{ij} = \frac{{A_{ij} }}{{\mathop \sum \nolimits_{j} A_{ij} }}$$

If the PageRank value of a node is large, it means that it is a high centrality node and it is probably a more suitable node to be selected as an effective node. By using the centrality criterion calculated in Eq. (7), the prominence of each node can be calculated based on the following equation:

15 $$P_{x} = \frac{1}{{\theta_{x} }}$$

By having the prominence criterion of each node by the above equation, it is possible to identify the leader nodes in the social network, which we will describe in the next section.

### Identification of influential nodes based on centrality and influence domain

Analyzing the behavior of users is one of the requirements to achieve the path of information dissemination in social networks. In every social group, some people have significant abilities in virtual communication. These people are connected to a wider part of society and can spread a large amount of information in a short period. These users are called influential users, and identifying them correctly can lead to access to information dissemination paths in the structure of social networks. An influential user attracts the attention of a relatively large group of social network users, and this group of users can be considered a community.

In the proposed method, an iterative algorithm is used to identify influential nodes in the social network. The suggested algorithm adopts the criteria of centrality and influence domain to identify this category of users and performs the identification process both at the community and network levels. To this end, assume a graph (or a subgraph of the social network that represents a community in the network structure) such as G. All connections in G are weighted using Eq. (2) and also the prominence of each node is calculated based on Eq. (15). The purpose of the algorithm used in this step is to estimate the attractiveness of each user for other social network users. For this purpose, the attractiveness model inspired by the firefly optimization algorithm in^20^ is utilized. In this model, the level of attractiveness of a user to others can be described as the following equation:

16 $$AT_{ij} = \beta \times e^{{ - a \times d_{ij}^{2} }} + P_{i}$$

**Figure 3:**
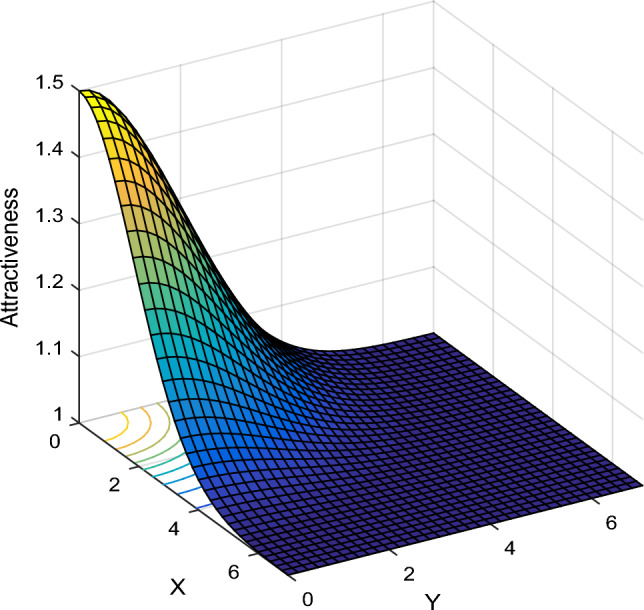
The attractiveness of a user with P_i_ = 1 for other users with different distances.

In the above relation, $AT_{ij}$ specifies the level of attractiveness of user i for user j. The parameter d_ij_ represents the shortest distance between users i and j, which is calculated based on the weighted graph (weighted according to Eq. (2)). Also, parameter β specifies the level of attractiveness in the distance d_ij_ = 0 and a indicates the rate of loss of influence of a user per unit of increase in distance. Finally, $P_{i}$ shows the prominence of user i, which is calculated based on Eq. (15). In fact, Eq. (16) is defined based on the concept of exponential reduction of a user’s influence in his domain and the distribution function of its values is shown in Fig. 3.

As shown in Fig. 3, the highest level of attraction of a user will be for people who have the smallest distance to that user. The users with the lowest distance are those who have the highest reaction rate (likes, reposts and comments) to the content generated by the user. Moreover, based on Eq. (16) and Fig. 3, if a user does not have any active communication, his/her attractiveness to other users will be equal to his/her prominence. This criterion can well describe the influence of a user on others. Nonetheless, it should be noted that to determine the influential nodes in social networks, in addition to the prominence criterion, the dispersion criterion should also be considered. This means that influential users are those who, in addition to being prominent, have a sufficient distance from other prominent people. With these explanations, the steps to identify effective users based on the attractiveness criterion will are given as follows:
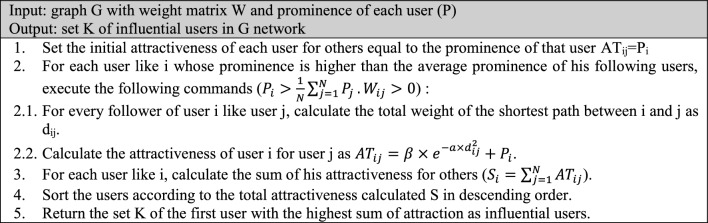


## Implementation and results

### Case I

**Figure 4:**
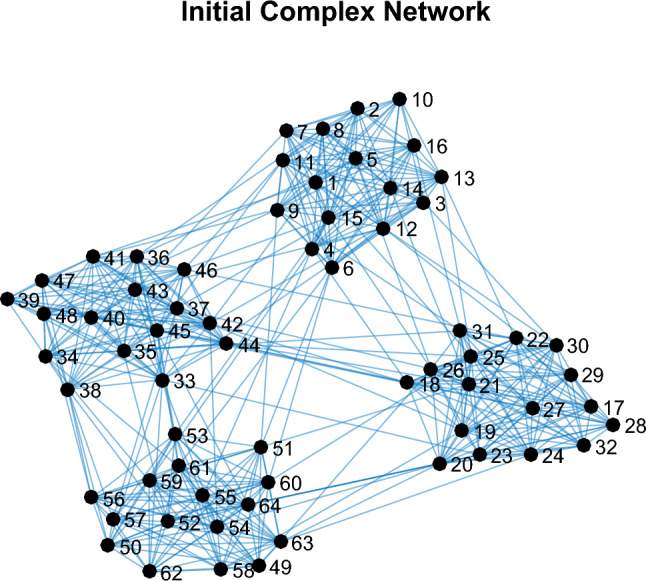
A hypothetical graph with 64 nodes and 5 clusters.

**Figure 5:**
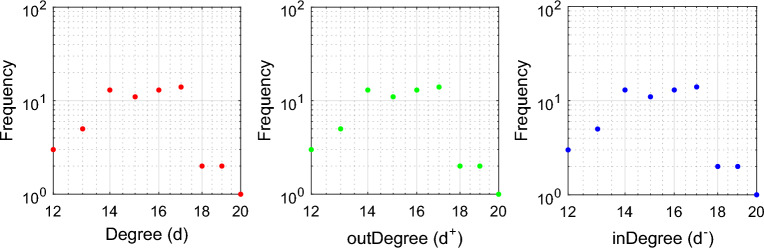
Distribution of overall degree (left), output degree (middle) and input (right) of network users in the previous figure.

The following presents the results of implementing the proposed method on a hypothetical network. A hypothetical network with 64 nodes and 4 communities (clusters) is considered. The structure of this social network is shown in Fig. 4. Also, in Fig. 5, the distribution of the overall degree, input degree and output degree of network users is displayed as a graph. Based on this figure, the degree of each user in the network is in the range^1,21^.

**Figure 6:**
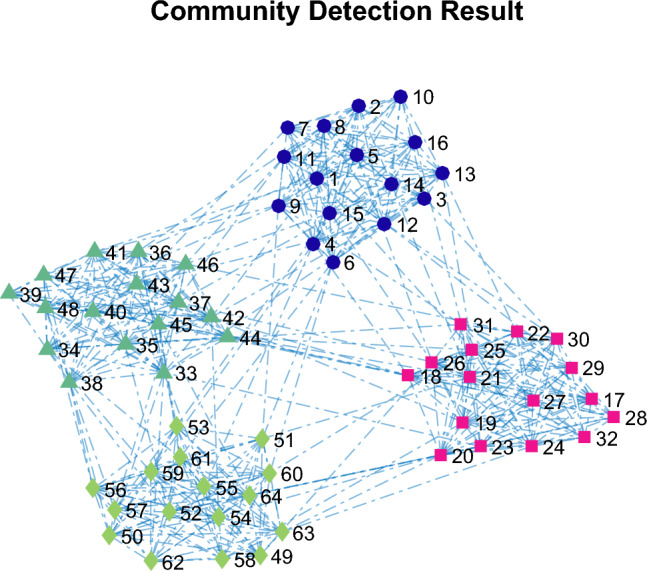
The result of graph clustering shown in Fig. 4.

**Figure 7:**
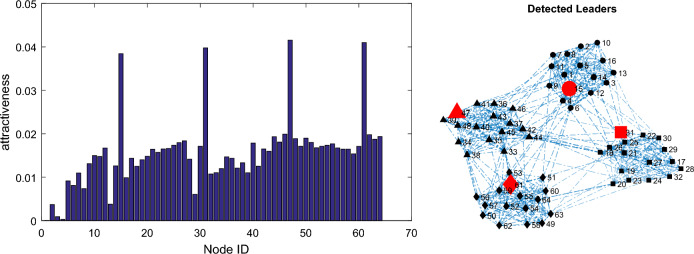
The degree of attractiveness of the nodes (left) and predicted leader nodes in the social network (right).

The social network is clustered using the Louvain algorithm. This algorithm is able to accurately detect the 4 communities in this graph and all the network nodes are correctly clustered, which results in achieving a value of 1 for the NMI criterion and 0.5288 for the modularity criterion in the clustered network. The clustering result is depicted in Fig. 6. Figure 7 illustrates the calculated attractiveness for each node based on the Firefly Optimization (FFO) algorithm^16^.

A leader node is extracted for each sub-community, but the main and overall community consists of several nodes. This issue is evident in Fig. 7, but it is possible to increase the number of leader nodes in the sub-community based on the attractiveness index, because the nodes are ranked based on the attractiveness index, and this issue is available in the paper.

### Case II

In this section, the implementation of the proposed method using MATLAB software has been explained and the performance of the method has been investigated. To evaluate the performance of the proposed method, the Twitter-Dynamic-Net database has been used^21^. This database includes 90,908 Twitter social network users with more than 443,000 time-based connections. In this database, there are more than 99 million data related to the behavior of users when facing the content of different users. Due to the large volume of this database, only information related to one year in this database has been used. Also, influential users have been identified in three 120-day periods. Thus, the database is sorted by time index and divided into three parts of 120 days. Next, the proposed method was applied to the accumulated data of each of these three intervals and the influential users of the social network were identified based on the time-based data. In other words, the process of detecting influential nodes by the proposed method has been iterated 3 times. In the first iteration, the data of the first 120 days of the year were used to identify the influential nodes by the proposed method, and in the second iteration, the data of the first 240 days of the year were used. Finally, all the data of the year have been used in the third iteration of the experiment. Each time the test is repeated, the performance of the proposed method has been evaluated in terms of correctness, accuracy, recall and processing time.

To evaluate the proposed method in terms of accuracy, we check the performance of the proposed method in selecting the correct influential nodes during different periods. If the number of social networks evaluation courses is equal to T, after each period such as $1 \le i < T$, the influential nodes in the social network are described as a set of network vertices such as $P_{i} \subseteq V$. This set is given as P_i_ = {v_1_,v_2_,…,v_x_}. Then, the list x of the nodes with the highest influence in the social network is extracted in the i + 1 period and it is shown as $\overline{{P_{i} }} \subseteq V$.

The presented method aims to select users as leader nodes (P_i_) that are as much as possible the same as the real leaders in the next interval ($\overline{{P_{i} }}$). This goal can be represented as a criterion such as $\frac{{\left| {P_{i} \cap \overline{{P_{i} }} } \right|}}{{\left| {\overline{{P_{i} }} } \right|}}$. By increasing the similarity of the list of recognized users and existing real leaders, this criterion becomes closer to 1, and by decreasing the similarity between these two sets, the value of this ratio approaches zero. In general, the relation $0 \le \frac{{\left| {P_{i} \cap \overline{{P_{i} }} } \right|}}{{\left| {\overline{{P_{i} }} } \right|}} \le 1$ will always be established. This ratio can be used as a criterion to evaluate the efficiency of the proposed method. Therefore, to evaluate the accuracy of the method, the following equation is used:

17 $$Acc = 100 \times \frac{1}{N} \mathop \sum \limits_{i = 1}^{N} \frac{{\left| {P_{i} \cap \overline{{P_{i} }} } \right|}}{{\left| {\overline{{P_{i} }} } \right|}}$$

where, *N* is the number of tests. Besides the above criteria, precision, recall and F-measure have also been used are criteria to investigate the performance of the suggested method. The precision specifies the proportion of correct outputs of the algorithm, which is calculated as follows:

18 $$precision = \frac{TP}{{TP + FP}}$$

where, TP represents the number of influential nodes in the social network that are correctly recognized by the proposed method. Also, FP indicates the number of users identified by the proposed algorithm as part of the influential users in the social network, but are not included in the set of real leaders in the next time frame.

The recovery criterion indicates the proportion of correct influential nodes that are correctly determined by the proposed algorithm. This criterion is calculated using the following equation:

19 $$recall = \frac{TP}{{TP + FN}}$$

where, FN represents the number of influential nodes that are in the set of real leaders in the next time frame, but the proposed method has not identified them as influential nodes in the latest time frame. Finally, using the above two criteria, the F-measure can be calculated as follows:

20 $$F{ - }Measure = \frac{{2 \times {\text{Precision}} \times {\text{Recall}}}}{{{\text{Precision}} + {\text{Recall}}}}$$

**Figure 8:**
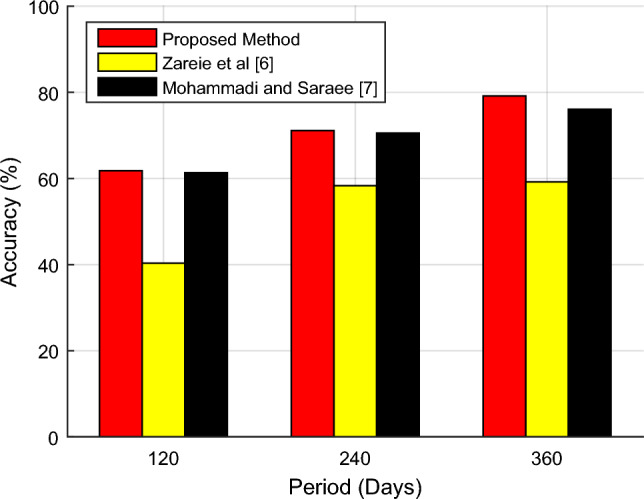
Changes in the accuracy of the proposed method in identifying influential nodes during different periods.

Figure 8 shows the accuracy changes of the proposed method and other compared methods in identifying the influential nodes during different time intervals.

**Figure 9:**
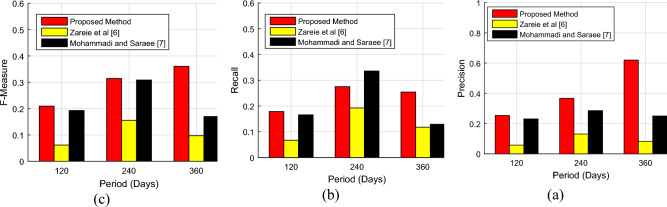
The values of (**a**) precision, (**b**) recall and (**c**) F-measure resulting from the detection of influential nodes by the proposed method during three 120-day periods.

As shown in Fig. 8, the accuracy of the proposed method in identifying influential nodes increases over time. The reason is that as time passes it will lead to the formation of a more complete model of the social network, and the proposed method can perform the identification process more accurately by using the total communication and reaction information of users more efficiently. The reaction information of users can be very effective in the more accurate identification of influential users, and the results presented in Fig. 8 confirm this point. Based on the results, the proposed method can finally achieve a detection accuracy of 79.15% using 360-day data, which shows an improvement of at least 3% compared to the previous methods. Meanwhile, in the first 120 days, the improvement achieved by the proposed method is only 0.5%. In this way, the proposed method can use reaction information more efficiently and increase the accuracy of identification to a greater extent. Also, Fig. 9 shows the values of precision, recall and F-measure resulting from the detection of influential nodes by the proposed method during three 120-day periods.

The results presented in Fig. 9 show that the proposed method can improve the precision, recall and F-measure criteria in the process of identifying influential users. These graphs also confirm that with more complete time-based information for each user, it is possible to predict the level of their influence on the social network with higher quality. For this reason, the graphs shown in Fig. 9 have a general upward trend. One can see carefully in Fig. 9a that the growth rate of the precision criterion over time is more than that of the recall criterion. This point demonstrates that with the increase in the volume of information, the proportion of correct outputs of the proposed algorithm will increase at a higher rate, and this feature shows the efficiency of the proposed method.

Over time, the underlying data distribution changes, which can affect the performance of machine learning models. Mohammadi and Saraei’s method in^7^ is more robust for moving data and has class imbalance, so it has better mechanisms to manage it, which may maintain higher recall. Mohammadi and Saraei’s algorithm specifically seeks to reduce the time of information dissemination, and this is achieved by several nodes; therefore, it identifies more nodes, and in some cases, it provides better performance over time. The model update frequency in Mohammadi and Saraei’s method includes frequent model updates or retraining, which can maintain higher recall by maintaining the latest trend of the data. In our proposed method, a set of k influential users (leaders) is returned by algorithms. Given that users are sorted by their attractiveness, if the value of k is large enough, the recall value will also be higher. If the value of k is small, the recall will also decrease because the number of results returned by the algorithm to the user depends on k. The closer the value of k is to the number of real leader users in the network, the higher the recall. Although the recall situation based on the technique proposed in^7^ is better than the suggested scheme in this work, according to Table 1, Ref.^7^ does not consider the prediction of the node. It did not include the topic of trust, but the proposed scheme has performed these studies.

In Table 2, the performance results of the proposed method in detecting influential users for three 120-day periods are displayed. Also, the results obtained by the proposed method have been compared with the methods presented in^5^ and^6^.

**Table 2:** The performance results of the proposed method in detecting influential nodes in different time frames.

		Proposed method	Zareie et al.; 2020^6^	Mohammadi and Saraee; 2018^7^
120 days	Precision	0.2537	0.0575	0.2307
	Recall	0.1786	0.0671	0.1655
	F-Measure	0.2096	0.0619	0.1927
	Accuracy	61.80%	40.35%	61.33%
	Time (s)	215.21	372.92	307.55
240 days	Precision	0.3668	0.1308	0.2857
	Recall	0.2752	0.1925	0.3358
	F-Measure	0.3145	0.1557	0.3087
	Accuracy	71.11%	58.33%	70.56%
	Time (s)	941.63	1606.48	1281.12
360 days	Precision	0.6202	0.0825	0.2500
	Recall	0.2542	0.1176	0.1290
	F-Measure	0.3606	0.0970	0.1702
	Accuracy	79.15%	59.20%	76.09%
	Time (s)	2135.44	3429.15	3260.01

As the comparisons in Table 2 show, the proposed method can identify influential users more accurately and efficiently. Also, the proposed method can perform network processing and identification on average in a shorter period. The improvement of accuracy and efficiency in the proposed method can be attributed to the use of the combination of communication and user reaction information, as well as the addition of link prediction. On the other hand, the use of network clustering and community identification solutions has reduced the complexity of the problem and identified social network users in a shorter period.

## Discussion and conclusion

In this article, a new method was presented to identify and predict influential nodes in the social network based on the structure of the local network communities and the reactive information of the users. The proposed method identifies the influencing nodes in five steps and is based on specific periods. The first step in the proposed method is to determine the importance of communication between users by calculating the weight of edges between network nodes. For this purpose, a criterion based on the rate of information dissemination and reactive behavior of users has been used. In the second step of the proposed method, the structure of the weighted network is decomposed into its constituent communities. The purpose of doing this work is to reduce the complexity of the problem of identifying the influential nodes through its analysis. After identifying the communities, link prediction is carried out based on the weighting of the previous stage and the amount of credit given to each node. In the fourth step, the centrality criteria are calculated for the members of each community to determine the importance of each node in the domain of its community. In the last step of the proposed method, an iterative algorithm is incorporated to identify influential nodes based on the centrality and influence domain of the node. The result of this algorithm is a set of influential users in the global domain of the network and the local domain of communities. The efficiency of the proposed method was evaluated using the Twitter-Dynamic-Net database and the results were compared with previous similar solutions. The results demonstrate that the suggested method can identify influential users with more accuracy, efficiency and speed. This performance improvement in the proposed method can be attributed to the adoption of the combination of communication and user reaction information. Additionally, using the network clustering solution and community identification reduces the complexity of the problem and identifies social network users in a shorter period.

In future works, the performance of multi-objective optimization algorithms can be studied for identifying the influential nodes in the last step of the method. Also, to further develop the proposed method, it is possible to investigate the application of other criteria based on centrality such as intermittent centrality and node mass to evaluate the prominence of social network users.

## Data Availability

All data generated or analysed during this study are included in this published article.
